# Monthly Record of Deaths in the City of Buffalo

**Published:** 1858-06

**Authors:** H. D. Garvin

**Affiliations:** Health Physician


### ART. IV.—*Report of Mortality in Buffalo for the month of April*, 1858.

By H. D. Garvin, M. D., Health Physician.

| DISEASES. | No. | Males. | Females. | No Sex given. |
|---|---|---|---|---|
| Abdominal Injury, | 1 | 1 | | |
| Cephalitis, | 1 | 1 | | |
| Child Birth, | 1 | | 1 | |
| Convulsions, | 15 | 5 | 7 | 3 |
| Croup, | 3 | 1 | 2 | |
| Debility, | 7 | 5 | 2 | |
| Dentition, | 1 | 1 | | |
| Diarrhœa, | 1 | | 1 | |
| Dysentery, | 1 | | 1 | |
| Dropsy, | 2 | 1 | 1 | |
| Drowned, | 1 | 1 | | |
| Eclampsia, | 1 | | 1 | |
| Epilepsy, | 1 | | 1 | |
| Exposure, | 1 | | 1 | |
| Fever, Typhoid, | 4 | 3 | 1 | |
| " Typhus, | 1 | 1 | | |
| Heart Disease, | 5 | 4 | 1 | |
| Hydrocephalus, | 4 | 2 | 2 | |
| Inflammation of Bowels, | 3 | 2 | 1 | |
| Injuries to Skull, | 1 | 1 | | |
| Jaundice, | 1 | 1 | | |
| Marasmus, | 5 | 2 | 3 | |
| Morbus Cerebis, | 1 | 1 | | |
| Meningitis, | 3 | 3 | | |
| Paralysis, | 2 | 2 | | |
| Pleuro-Pneumonia, | 1 | 1 | | |
| Pneumonia, | 5 | 2 | 3 | |
| Phthisis, | 16 | 11 | 2 | 3 |
| Puerperal Fever, | 2 | | 2 | |
| Pulmonary Congestion, | 2 | 2 | | |
| Premature Birth, | 1 | | 1 | |
| Psoriasis, | 1 | | 1 | |
| Pulmonary Apoplexy, | 1 | | 1 | |
| Rheumatism, | 1 | | 1 | |
| Rubeola, | 1 | | 1 | |
| Scarlatina, | 2 | 2 | | |
| Smothering, | 1 | | 1 | |
| Still Born, | 2 | 1 | 1 | |
| Scrofula of Knee, | 1 | 1 | | |
| Typhoid Pneumonia, | 1 | | 1 | |
| Tumor of Kidney, | 1 | | 1 | |
| Uterine Cancer, | 1 | | 1 | |
| Total, | 124 | | | |

### SEXES.

| | |
|---|---|
| Males, | 58 |
| Females, | 43 |
| Sex not given, | 23 |
| Total, | 124 |

AGES.

| | | | |
|---|---|---|---|
| Still-born, | 2 | Between 20 years and 30 years, | 8 |
| 1 day, | 3 | " 30 " " 40 " | 8 |
| 1 day and 30 days, | 10 | " 40 " " 50 " | 5 |
| Between 1 month and 6 months, | 15 | " 50 " " 60 " | 11 |
| " 6 months and 12 " | 12 | " 60 " " 70 " | 2 |
| " 1 year " 3 years, | 21 | " 70 " " 80 " | 6 |
| " 3 " " 5 " | 6 | " 80 " " 90 " | 1 |
| " 5 " " 10 " | 1 | " 90 " " 100 " | 0 |
| " 10 " " 20 " | 7 | " 100 " | 0 |
| | 77 | | 41 |
| Ages not given, | 6 | | 118 |
| Total, | 124 | | |

NATIVITIES.

| | | | |
|---|---|---|---|
| American, | 83 | Prussian, | 0 |
| German, | 25 | Italy, | 1 |
| Irish, | 11 | French, | 1 |
| English, | 0 | Scotch, | 0 |
| Canadian, | 0 | Bohemia. | 0 |
| Holland, | 0 | Country not given, | 3 |
| Total, | | | 124 |

